# Health care workers’ need for support from managers during the initial phase of the COVID-19 pandemic

**DOI:** 10.1186/s12913-022-08937-9

**Published:** 2022-12-21

**Authors:** Matilda Skogsberg, Gustav Jarl, Marie Matérne

**Affiliations:** 1 Regional Health, Region Örebro County, Örebro, Sweden; 2 University Health Care Research Center, Faculty of Medicine and Health, Örebro University, Örebro, Sweden; 3 Department of Prosthetics and Orthotics, Faculty of Medicine and Health, Örebro University, Örebro, Sweden; 4 School of Law, Psychology and Social Work, Örebro University, Örebro, Sweden

**Keywords:** COVID-19 pandemic, Health care workers, Support, Manager, Leadership, Mental health

## Abstract

**Background:**

The COVID-19 pandemic placed great pressure on health care workers and increased the risk of these workers developing mental illness. Effective leadership is essential to prevent mental illness from developing. The study aim was to investigate health care workers’ perceptions of the support given by their managers, their need for such support, and their levels of anxiety during the initial phase of the COVID-19 pandemic.

**Methods:**

An electronic study-specific questionnaire was sent to health care workers. A convergent mixed-methods design was used, in which data were analyzed with descriptive statistics, t-tests, and qualitative content analysis.

**Results:**

The majority of the 1074 participants experienced support from a manager who was physically present, caring, and sensitive to their needs, who provided information, and listened to their opinions. Those who did not receive this support experienced more anxiety, and the majority of them expressed a need for this support. A minority of the participants had a manager who scheduled reflection time; few asked for this support, and it was not found to be associated with lower anxiety levels. The qualitative analysis resulted in four themes: 1) A physically present and responsive manager who provides support based on the workers’ needs, 2) The workers understand their manager’s tough work situation and lack of resources, 3) There is a need for clear dissemination of information and participation in organizing the work, and 4) The care of patients is carried out with good cooperation.

**Conclusions:**

The majority of the health care workers received adequate support from their managers. A manager has to be physically present, caring, and supportive to meet the workers’ needs and potentially reduce anxiety levels.

## Background

The coronavirus disease COVID-19 was first identified in December 2019 in Wuhan, China, and it rapidly spread to the rest of the world [1]. In January 2020, the first case of COVID-19 was diagnosed in Sweden [2]. Community transmission was confirmed in March 2020. During this initial phase the Swedish health care system had to reorganize to expand health care service capacity and reassign health care workers to provide care and treatment for COVID-19 infected patients [3]. Patients were treated within hospital care, primary care, and municipal care all over the country. In the county of Örebro, where the study took place, there were 63-78 COVID-19 inpatients per day and 245 infected residents treated in municipal care [4]. The challenges were many; increased workload, fear of getting infected, working with protective equipment, new routines, working with seriously ill patients whose condition could deteriorate quickly. In several cases, resources were used to their limit and difficult decisions needed to be made. In some cases, health care workers worked long shifts and had less time for recovery [5]. High demands in a work environment is a risk factor in terms of mental health [6]. A pandemic is not an exception, the great pressure on the health care workers increased the risk of developing mental illness such as stress, anxiety and depression[1, 7, 8].

Many health care organizations provided support to promote health and well-being among health care workers during the pandemic [5]. Various support measures have been described and recommended. For example, support to meet basic needs, such as sleep, food, recuperation, and social contacts [9] or to receive training in paying attention to symptoms of stress, using coping strategies, and getting professional help when needed [10]. Several studies have also recommended that organizations create different support systems, for example, networks with peer support or regular meetings to discuss difficult clinical decisions and situations [5, 10–14]. Others have placed greater emphasis on support efforts at the organizational level and, among other things, recommended a visible and clear leadership that is sensitive to the health care workers’ needs [5, 13, 15]. The importance of leadership was further supported by a systematic review of 31 articles, where the authors concluded that effective leadership is vital to prevent mental illness among health care workers during the pandemic [16].

Leadership during normal circumstances can be difficult, leadership during a pandemic is even more complex [5]. Managers need to handle the substantial challenge in implementing strategies directed from above, and support subordinates during difficult times and at the same time being equally impacted by the pandemic as the employees [17].

For employees managers are seen as an important source of support at work [18]. There is, however, a lack of studies about health care workers’ self-reported support needs and experiences of the support they have received. In the absence of this information, the support provided may not match the needs of these workers. For instance, a detailed plan was initially developed in China to prevent mental illness among health care workers but later had to be adjusted because studies showed that it did not match the needs of the workers [9].

The purpose of the study was to investigate health care workers’ perceptions of the support given by their managers, their need for support from their managers, and their levels of anxiety during the initial phase of the COVID-19 pandemic.

## Methods

The study had a convergent mixed-methods design [19] where qualitative and quantitative data were collected from the same study sample in a cross-sectional survey.

### Participants and procedure

The study participants were health care workers who worked during the initial phase of the COVID-19 pandemic (13 April – 6 May 2020) with patients in Örebro County, Sweden, who were infected or suspected to be infected with COVID-19. The settings included hospital care (COVID-19 intensive care unit, COVID-19 wards and emergency department), primary care, and municipal care. All the hospital departments (except the emergency department), 17 out of 29 health care centers and the municipal care approved the distribution of the survey. An estimated 3000 questionnaires were sent out, 1278 in hospital care, 879 in primary care while the numbers within the municipal care is unclear.. The questionnaire was created using the survey platform esMaker (https://www.entergate.se/esmaker) and distributed online from the researchers via health care managers to the workers during May and June 2020, with two reminders. The study was approved by the Swedish Ethical Review Authority (Reg.no. 2020-01784). Informed consent was obtained by virtue of them having responded.

### Questionnaire

A study-specific questionnaire was constructed to assess the health care workers’ experiences of anxiety and its possible impact on work ability, ethical stress, and various types of support. This study focused on support from manager and included the following seven items from the questionnaire: 1) My manager is visible and physically present and asks how it is going, 2) My manager provides clear and coordinated information, 3) My manager provides advice and help with priorities in the care of patients, 4) My manager is sensitive to my needs (e.g. how I feel, my schedule, recuperation, security), 5) My manager listens to me and takes me seriously regarding my opinions about the organization of the work, 6) My manager schedules reflection time (e.g. ‘after-action reviews’) at the end of the work shift (What did we do well and why? What did we do less well and how should we change it?), and 7) Do you want other support from your manager? Each of the items 1–6 had three response alternatives [Yes; No; Don’t know] and was followed by a question to be answered by those who answered No: If not, do you need this? [Yes; No]. There was also a box for comments following each of the first six items. Item 7 had no pre-specified response alternatives, but only a box for open responses.

In addition to the questionnaire, participants completed the Generalized Anxiety Disorder 7-item scale (GAD-7), which measures generalized anxiety on a four-category rating scale (from 0 = “Not at all” to 3 = “Nearly every day”). Higher scores indicate higher levels of anxiety where 0-4 points are interpreted as minimal anxiety, 5-9 points as mild, 10-14 points as moderate and ≥15 points as severe anxiety [20].

### Analysis

Quantitative and qualitative data were analyzed separately. Thereafter the results were considered as a whole.

For the quantitative analysis, we used the answers to items 1–6. For each item, responses to the first part (support provided) and follow-up question (support needed) were combined into four response categories [Yes; No, I need this; No, I don’t need this; Don’t know]. The answers were summarized with descriptive statistics. The mean values on GAD-7 were compared between people who received and did not receive each type of support, by calculating 95% confidence intervals based on the t-test. IBM SPSS Statistics version 25.0 was used for all quantitative analyses (Armonk, NY: IBM Corp.).

For the qualitative analyses, we used the comments on items 1–6 and the open-ended answers on item 7. The comments were analysed using tables in Microsoft Word software. For this data, a qualitative content analysis with an inductive approach was used. In an inductive analysis, the researcher strives to be open-minded and the analytic process is data-driven, as opposed to in a deductive analysis where the analytic process is theory-driven. The content analysis consisted of seven steps [21]. The first and second step included finding meaningful parts of the text and condensing them into meaningful units. In this study these two steps were merged, since each comment was considered to be a meaningful unit. In the third step, the meaningful units were labeled with a code that briefly described its content. In the fourth step, codes with similar content were formed into subcategories. In the fifth step, these subcategories were combined into categories. Until this step in the process, the items were analyzed separately. In the sixth step, all data were put together to create themes which contain the underlying meaning of several categories. This analysis process was conducted by the first author in close dialog with the last author, but the two authors analyzed the data individually before they discussed the results to reach consensus. In the last step, the final result was discussed with the second author to reach an agreement and make decisions on which themes best represented the data.

## Results

A total of 1074 participants were included in the study, which corresponds to a response rate of approximately 35%. The occupational categories that were most prevalent were nurses, assistant nurses, doctors, and physiotherapists. They accounted for 86% of the participants. Examples of occupational categories that occurred to a lesser extent were care assistants, occupational therapists, counselors, housing support officers, and midwives. Participants worked with COVID-19 patients at their regular workplace or had been referred to or volunteered to work at another workplace with COVID-19 patients. The majority, 78%, worked in Region Örebro County in units such as intensive care, COVID wards, athirdsecond author to reach annd primary care. The other 31% worked in nursing homes for the elderly in the county’s municipalities. The reason why the total number exceeded 100% was that 95 participants stated that they worked at more than one workplace. To ensure anonymity demographic data on age, gender, and work experience were not collected for ethical reasons.

### Quantitative results

The responses to the first five items (the manager is visible, provides information, provides advice, is sensitive to needs, and listens) shared a similar pattern of responses: 51.2%–73.2% answered Yes; 10.5%–17.2% answered No, I need this; and 0.7%–13.1% answered No, I don’t need this (Table 1). The response pattern for the sixth item (whether the manager schedules reflection time) was distinct from the other questions. This item had the lowest proportion of participants who answered Yes (17.7%) and the highest proportion of participants who answered No, I need this (18.4%) or No, I don’t need this (45.0%).

**Table 1:** Descriptive statistics for the multiple-choice items (*n*=1074)

1. My manager is visible and physically present and asks how it is going.	N (%)
Yes	786 (73.2)
I don’t know	103 (9.6)
No, but I need this	143 (13.3)
No, I don’t need this	42 (3.9)
2. My manager provides clear and coordinated information.	N (%)
Yes	780 (72.6)
I don’t know	112 (10.4)
No, but I need this	175 (16.3)
No, I don’t need this	7 (0.7)
3. My manager provides advice and help with priorities in the care of patients/residents.	N (%)
Yes	550 (51.2)
I don’t know	269 (25)
No, but I need this	114 (10.6)
No, I don’t need this	141 (13.1)
4. My manager is sensitive to my needs (e.g. how I feel, my schedule, recuperation, security).	N (%)
Yes	681 (63.4)
I don’t know	187 (17.4)
No, but I need this	185 (17.2)
No, I don’t need this	21 (2.0)
5. My manager listens to me and takes me seriously regarding opinions about the organization of work.	N (%)
Yes	700 (65.2)
I don’t know	248 (23.1)
No, but I need this	113 (10.5)
No, I don’t need this	13 (1.2)
6. My manager schedules reflection time (e.g. “after-action review”) at the end of the work shift. (*What did we do well and why? What did we do less well and how should we change it?)*	N (%)
Yes	190 (17.7)
I don’t know	203 (18.9)
No, but I need this	198 (18.4)
No, I don’t need this.	483 (45.0)

On the first five items, about support received from the manager (the manager is visible, provides information, provides advice, is sensitive to needs, and listens), participants who answered No scored on average 2.8–3.4 points higher on GAD-7 than those who answered Yes (Table 2). These differences were statistically significant and indicate that people not receiving these types of support had a higher level of anxiety. On the last question, whether the manager schedules reflection time, the difference in GAD-7 scores between people answering No and Yes was small, 0.6 points, and not statistically significant.

**Table 2:** Comparison of scores on the Generalized Anxiety Disorder 7-item scale (GAD-7) between participants reporting and not reporting support from their manager (*n*=1074)

	***GAD-7, mean (SD)***		***Difference, No minus Yes (95% CI) ***^***a***^
	**Yes**	**No**	
1. My manager is visible and physically present and asks how it is going.	4.0 (4.7)	6.9 (5.7)	2.9 (2.0 to 3.8)
2. My manager provides clear and coordinated information.	4.0 (4.7)	7.2 (5.6)	3.1 (2.2 to 4.0)
3. My manager provides advice and help with priorities in the care of patients.	3.7 (4.5)	6.5 (5.6)	2.8 (2.0 to 3.6)
4. My manager is sensitive to my needs (e.g. how I feel, my schedule, recuperation, security).	3.9 (4.7)	7.3 (5.8)	3.4 (2.5 to 4.3)
5. My manager listens to me and takes me seriously regarding opinions about the organization of work.	4.0 (4.6)	7.2 (5.9)	3.2 (2.1 to 4.3)
6. My manager schedules reflection time (e.g. “after-action review”) at the end of the work shift. (*What did we do well and why? What did we do less well and how should we change it?)*	4.3 (5.0)	4.9 (5.1)	0.6 (-0.2 to 1.4)

*CI* Confidence interval

^a^For items 1–5, Leven’s test had *p*<0.05, therefore, t-test not assuming equal variances was used. For item 6, Leven’s test had *p*=0.524, therefore t-test with equal variances assumed was used

### Qualitative results

The open response options generated 1464 comments. The analysis of these comments resulted in 13 categories and four themes related to support from managers (Table 3).

**Table 3:** Results from qualitative analysis of the comments and the open-ended question “Do you want other support from your manager?”

Categories	Themes
1. A manager who is physically present. 2. A manager who understands and listens to the workers’ needs. 3. A manager who provides support and information. 4. A manager who has competence.	A physically present and responsive manager who provides support based on the workers’ needs
1. The manager has an unclear role and a tough work situation. 2. The workers understand the manager’s work situation. 3. The manager listens to opinions but lacks resources for making changes.	The workers understand their manager’s tough work situation and lack of resources.
1. The dissemination of information is of varying quantity, quality, and clarity. 2. The workers want to be involved and participate in organizing the work.	There is a need for clear dissemination of information and participation in organizing the work.
1. Organizing the work takes place through dialogue in the workplace. 2. Different professions are responsible for making priorities. 3. Other methods for prioritizing are available. 4. Time for reflection is available when needed.	The care of patients is carried out with good cooperation.

Four themes were identified related to what support the health care workers received and needed from their managers: 1) A physically present and responsive manager who provides support based on the workers’ needs; 2) The workers understand their manager’s tough work situation and lack of resources; 3) There is a need for clear dissemination of information and participation in organizing the work; 4) The care of patients is carried out with good cooperation.

The results are presented below theme by theme, with the categories indicated.

#### A physically present and responsive manager who provides support based on needs

This theme included the following categories: A manager who is physically present; A manager who understands and listens to the workers’ needs; A manager who provides support and information; and A manager who has competence.

During an uncertain period with limited knowledge about the new coronavirus, the health care workers were in an exposed situation. The results show that they needed a manager’s physical presence and support during this time. Most participants reported that their manager was available, either in person or reachable by phone and text messages. Most had daily contact with their manager. The managers were also described as committed and approachable, that they cared about the health care workers, asked how they were doing, and listened and gave them support. One comment was:

“We have had a fantastic manager, always supported us! Came up several times during the work shift to check how we were doing, how we experienced everything and if we had any questions." [Id 310, assistant nurse, municipal care]

The participants appreciated their manager’s efforts. They considered the support given to be adequate and reported that it made a difference. They felt they had a manager they could trust. The following quote highlights this:

“Our manager is a rock we cannot be without. She sees, hears, communicates and keeps track. She is what a manager should be.” [Id 3, assistant nurse, intensive care unit]

For some of the participants, the manager was not seen at the workplace, and they had to search for him or her if they needed support. They expressed a wish for a manager who was physically present and aware of what was happening at the workplace. The participants also expressed the need to be seen individually, to get encouragement, or to get help when not feeling well. One participant described it as follows:

“Our manager has been really invisible and has mostly worked from home. Updated us very poorly through email etc." [Id 213, registered nurse, municipal care]

Moreover, some participants commented that their managers had limited competence and did not understand their situation. Several described their situation as disorganized. A new disease meant changes in how work was organized and performed. There was a constant flow of information and rapid changes. For many, this was a mentally stressful situation. The participants observed that managers were poorly acquainted with various routines and working methods. The participants expressed, among other things, the need for regular meetings to follow up the work according to the new guidelines. Some felt that their manager did not take the workers’ difficulties and concerns seriously. One comment was:

“.. however, I did not feel that the managers had a full understanding of how it was for us who were with the patients and how much resources we required with each COVID-19 patient.” [Id 1046, registered nurse, COVID-19 ward at hospital]

#### The workers understand their manager’s tough work situation and lack of resources

The second theme included the following three categories: The manager has an unclear role and a tough work situation; The workers understand their manager’s work situation; and The manager listens to opinions but lacks resources for making changes.

It emerged that there was a need for support from the manager, but the participants felt that their managers had limited ability to provide that support. Among other things, the participants attributed this to the managers having a lot to deal with. The participants considered their managers to be under pressure, busy, overloaded, or stressed. One comment was:

“Some support, but I think we are all in an extremely stressful situation. But email conversations concerning work are going on so you notice that they [the managers] are working hard too. But no one directly asks how you feel and how things are going.” [Id 112, registered nurse, intensive care unit]

A number of comments were about unclear leadership. Some participants had several managers with different areas of responsibility, which made it hard to know who to turn to. For some, who had all temporarily changed workplace, it was unclear who their immediate manager in charge was. One comment was:

“Many different managers. Hard to know who does what. One Heroma [salary system for human resources] manager, one operations manager, one health care manager. It’s a mess!” [Id 5, registered nurse, intensive care unit]

There were also factors that the managers could not control, which gave them limited options to take action. Examples of such factors were a lack of available information, changed conditions, and decisions that the manager did not have the mandate to influence. One participant described it as follows:

“Yes, but it isn’t easy for our immediate managers because the conditions are constantly changing.” [Id 13, assistant nurse, intensive care unit]

A recurring problem that became apparent was the lack of adequate recuperation. It was difficult to create a time schedule in which the health care workers got enough recuperation between working shifts. In order to be able to staff all shifts, some had to work more than usual, either longer shifts or more shifts than their ordinary schedule. According to the participants, the organization’s need for staff took precedence over the need for recuperation. Some described signs of mental ill health in the form of stress and difficulties sleeping. It was also difficult to get leave days granted. This meant that health care workers lived in uncertainty about getting leave days, which in itself created stress. One participant described it as follows:

“There is no consideration for rest and recuperation due to the tough work environment. No understanding of the psychological strain you are exposed to.” [Id 60, registered nurse, “other” workplace at hospital]

Throughout, there was an understanding of the manager’s limited options. In some cases, the participants felt that they could still receive some support, in other cases there was no support. One comment was as follows:

“Feel that the management has done the best they could do and had time for.” [Id 62, assistant nurse, COVID-19 ward at hospital]

#### There is a need for clear dissemination of information and participation in organizing the work

The third theme included two categories: The dissemination of information is of varying quantity, quality, and clarity and The workers want to be involved and participate in organizing the work.

There was an overwhelming information flow during the initial phase of the pandemic, and the health care workers needed help to filter and sort the information. The results showed that in some cases there was too much information for them to handle and relate to. In other cases, they experienced a lack of information, either that the information was incomplete or that it did not reach everyone involved. Further, there were different channels for disseminating information. Here, the participants usually valued oral information face to face, for example at meetings, to have the chance to ask questions and get clarifications. However, a lot of information was disseminated either by email, websites, or through new routines. Sometimes a huge responsibility was placed on the health care workers to access the necessary information themselves to stay updated. One participant described it as follows:

“Around 60 emails a week from different managers with different information. You don’t have time to get acquainted with everything new.” [Id 170, doctor, COVID-19 ward at hospital]

Furthermore, difficulties arose regarding the fact that the information was sometimes unclear or changed at short notice, that it was not coordinated between different managers, and that managers sometimes sent conflicting messages. Several participants described how the lack of clear information could create rumors and misunderstandings that made it difficult for the health care workers to do their job. One comment was:

“We do get information, but sometimes we get the same information by email from several sources. In addition, information and routines change from morning to afternoon and from day to day, so sometimes you don’t know what applies if you have been absent.” [Id 669, “other” profession, primary care]

The participants also felt that their views and suggestions for improvements in the organization of patient care were not taken seriously. For example, the manager made a decision without listening to the health care workers or the manager listened but it did not lead to any change. The participants expressed a desire to be more involved in organizing the work. There was a request for openness to express different points of view and to be able to have a dialogue about what would be the best care for the patients. One comment was:

“Our manager rarely takes what we say seriously or is interested in trying to understand what problems exist and how we can help each other to improve.” [Id 563, assistant nurse, “other” workplace at hospital]

#### The care of patients is carried out with good cooperation

This last theme included the following four categories: Organizing the work takes place through dialogue in the workplace; Different professions are responsible for making priorities; Other methods for prioritizing are available; and Time for reflection is available when needed.

It emerged that these health care workers had found working methods that were successful and that participation and communication worked satisfactorily on several levels in the organization. The participants described managers who listened and took their opinions seriously when it came to organizing the work, and that the workers were involved in decision-making. One participant described it as follows:

“We have worked together in the department and jointly built it up. We help each other and everyone can come forward with opinions and make themselves heard.” [Id 66, assistant nurse, COVID-19 ward at hospital]

When it came to prioritizing, there were routines that worked well. For example, certain professions were responsible for making priorities, or the priorities were made in dialogue with the other professions, and the manager gave advice and help when needed. Two comments were:

“Very clear, good manager, but priorities in care are managed by the reliably attending and participating anesthetists, not by her.” [Id 672, registered nurse, “other” workplace at hospital]

“We have a dialogue in the team – manager, nurse, occupational therapist, assistant nurse – where we discuss how to prioritize.” [Id 920, assistant nurse, municipal care]

When needed, there were opportunities for collaborative reflection during working hours. It could be that reflection time was scheduled, that it was scheduled when needed, or that there was time for reflection during the work. One participant described it as follows:

“We have time for joint reflection during work shifts and during shift changes and handovers.” [Id 784, assistant nurse, municipal care]

## Discussion

The purpose of the study was to investigate the health care workers’ perceptions of the support given by their managers, their need for support from their manager, and their levels of anxiety during the initial phase of the COVID-19 pandemic. We found that the majority of the participants felt that their managers provided support by being physically present at the workplace and asking how the work is going, providing clear and coordinated information, being sensitive to their needs and listening to their opinions. In the cases where the manager did not provide this support, anxiety levels were higher, and the majority of the participants expressed a need for this support. It became clear from the results that this kind of support from managers was important, that it genuinely made a difference for the health care workers during a time with many challenges; a new disease, lack of knowledge, and constant changes in how the work was organized and performed. The COVID-19 pandemic put intense pressure on the health care workers’ ability to process information, make assessments and decisions, hide their emotional reactions, contain feeling from others, and deal with ethical dilemmas [4]. To handle all these cognitive and emotional demands, the health care workers needed a physically present manager who understood, supported, and cared for them. They needed someone with the ability to create stability and involvement among the health care workers in a time of uncertainty and disorganization.

Other studies focusing on the health care workers’ experiences and their need for support from managers during the COVID-19 pandemic have come to similar conclusions: the workers need a manager who is physically present, listening, caring, and communicating [22–24]. Shanafelt et. al. [24] emphasize a visible leadership, which resonates with our study participants’ expressed need for a manager who is physically present. A manager who is on site is a prerequisite for the support they wished for. This allows the manager to be attentive to the health care workers’ concerns and well-being and to meet any individual needs that might exist. A physically present manager in the workplace is also able to provide the health care workers with adequate information and to have a dialogue based on this information when needed. It enables the manager to listen to the health care workers’ opinions and involve them in the organization of their work.

We found that about half of the participants had a manager who provided advice and help with priorities in the care of patients. Of those who did not have a manager who provided this help, about half thought it was needed. The participants thought that the care of patients was carried out with good cooperation, both between the managers and the health care workers, and between colleagues. Overall, the majority of the participants had good support from the manager and, even though there were needs that were not met, the participants understood the manager’s limited possibilities to solve all problems.

One need that was noticeably less important than the others for our participants was time for collaborative reflection. Only about a fifth of the participants had a manager who scheduled time for reflection at the end of the work shift. Almost half of the participants did not think this was needed (45%), while approximately one third of those who did not have this support did express a need for it (18.4% of all participants). Moreover, those who had this support did not report less anxiety on GAD-7 than those without this support. A possible explanation for the different results for this type of support may be that collaborative reflection might not be necessary when there is good support from the manager and the care of patients is carried out with good cooperation. If the dialogue needed is possible during work shifts, no extra time for reflection needs to be scheduled.

Support from managers is of great concern for the health care workers’ own health. This can be illustrated by the Job–Demand–Control–Support model [25]. The model shows how job demands can cause mental illness if there is not enough support.

Support from the immediate manager in the form of care, encouragement and feedback, information, and concrete practical help is important to reduce the risk of mental illness. Several studies have confirmed this during the pandemic. An American study [26] concluded that lack of leadership support was one of the factors that were most strongly associated with the experience of symptoms such as PTSD, major depressive disorder, and generalized anxiety disorder among frontline health care workers. Furthermore, an Australian study [27] found that health care workers’ risk of experiencing anxiety, depression, burnout, and PTSD was decreased by half when they felt supported by their organization. Our results add to this evidence, showing that people who receive support from their managers experience less anxiety compared to those without this support. Therefore, if health care managers and organizations provide adequate information, communication, and support, it seems likely that some of the burden on health care workers’ mental health could be reduced during the initial phase of a large crisis, such as a global pandemic [28].

### Limitations

At the time when the questionnaire was sent out, the situation was new and unpredictable. Knowledge about the virus and the treatment for infected patients was poor, but decision-making was fast, and adjustments and reorganization of the work routines were made continuously. In a rapidly evolving situation, the need for support from managers might change. Since our results reflect the participants’ experiences during the initial phase of the pandemic, they may not be generalizable to later phases of the pandemic. Furthermore, given the cross-sectional and observational study design, causal inferences between perceived support and anxiety levels should be drawn with caution.

The study focused on experiences among health care workers in a limited geographical area in Sweden. Support from managers may vary between individual managers, health care systems, cultures, and so on, which limits the generalizability of the result. Regarding what kind of support health care workers need, the results from this analysis match results from earlier finding in other countries [22–24] and are therefore likely more generalizable.

In addition, the question regarding the need for each particular type of support was only posed to people who answered that they had not received that support. Thus, we cannot know whether participants who had received the support perceived it to be needed or not.

### Future research

It would have been interesting to conduct interviews to get a deeper understanding of the support needed, as well as a follow-up study to see if the perceptions of support given and support needed have changed during the pandemic. Interventional studies are also needed to see whether enhanced support from managers improves the mental health of health care workers. Furthermore, it would be interesting to investigate what support the managers have experienced during the pandemic, since their well-being is a prerequisite to be able to support the health care workers [29].

## Conclusions

Support from managers is of great concern for health care workers’ mental health and performance during a pandemic. The most valued support that emerged from this study is a physically present manager who cares about the health care workers’ well-being, provides clear and coordinated information, is sensitive to their needs, and listens to their opinions. This support is associated with lower levels of anxiety among the health care workers in this study. The majority of the participants felt that they had this support, even though they wished it could be met to a greater extent. Another valued improvement was to be involved in how the work was organized, which is an important lesson to be transferred in the case of future challenges to health care organizations.

## Data Availability

The datasets generated and/or analysed during the current study are not publicly available due to current Swedish ethical legislation and European union GDPR act, but are available from the corresponding author on reasonable request, if appropriate permits are obtained from adequate authorities.
